# Non–*C. difficile* *Clostridioides* Bacteremia in Intensive Care Patients, France

**DOI:** 10.3201/eid2707.203471

**Published:** 2021-07

**Authors:** Guillaume Morel, Guillaume Mulier, Etienne Ghrenassia, Moustafa Abdel Nabey, Yacine Tandjaoui, Achille Kouatchet, Laura Platon, Frédéric Pène, Anne-Sophie Moreau, Amelie Seguin, Damien Contou, Romain Sonneville, David Rousset, Muriel Picard, Guillaume Dumas, Djamel Mokart, Bruno Megarbane, Guillaume Voiriot, Isabelle Oddou, Elie Azoulay, Lucie Biard, Lara Zafrani

**Affiliations:** Hautepierre Hospital—University Medical Center, Strasbourg, France (G. Morel);; Hospital Saint Louis, APHP, Paris, France (G. Morel, G. Mulier, E. Ghrenassia, M.A. Nabey, E. Azoulay, L. Biard, L. Zafrani);; Hospital Avicenne, Bobigny, France (Y. Tandjaoui); Teaching Hospital, Angers, France (A. Kouatchet);; Lapeyronie University Hospital, Montpellier, France (L. Platon);; Hospital Cochin, APHP, Paris (F. Pène);; Salengro Hospital, Lille, France (A.-S. Moreau);; University Hospital, Nantes, France (A. Seguin);; Victor Dupouy Hospital, Argenteuil, France (D. Contou); Hospital Bichat, APHP, Paris (R. Sonneville);; University Teaching Hospital, Toulouse, France (D. Rousset),; Institut Universitaire du Cancer—Oncopole, Toulouse (M. Picard);; Hospital Saint Antoine, APHP, Paris (G. Dumas);; Paoli-Calmettes Institute, Marseille, France (D. Mokart);; Hospital Lariboisiere, APHP, Paris (B. Megarbane);; Hospital Tenon, APHP, Paris (G. Voiriot);; University Medical Center, Strasbourg (I. Oddou);; INSERM U976, Paris (L. Zafrani)

**Keywords:** *Clostridium*, *Clostridioides*, bacteremia, ICU, intensive care, hemolysis, bacteria, France

## Abstract

Usually responsible for soft tissue infections, *Clostridioides* species can also cause bacteremia, life-threatening infections often requiring intensive care unit (ICU) admission. We conducted a multicenter retrospective study to investigate *Clostridioides* bacteremia in ICUs to describe the clinical and biologic characteristics and outcomes in critically ill patients. We identified 135 patients with *Clostridioides* bacteremia, which occurred almost exclusively (96%) in patients with underlying conditions. Septic shock and digestive symptoms were the hallmarks of *Clostridioides* bacteremia in the ICU. We identified 16 different species of *Clostridioides*, among which *C. perfringens* accounted for 31% of cases. Despite the high sensitivity of *Clostridioides* to common antimicrobial drugs, mortality rates were high: 52% for ICU patients and 71% overall at 3 months. In multivariate analysis, the most important factor associated with increased risk for death was the presence of hemolysis. *Clostridioides* bacteremia often leads to multiple organ failures, which have high mortality rates.

## Abstract 2

Article Summary: This multicenter study focusing on critically ill patients showed a strong relationship between hemolysis and mortality.

Obligate anaerobic bacteremia is a rare event, accounting for ≈0.1%–10% of positive blood cultures; *Clostridioides* spp. bacteremia represents 8%–46% of the cases (*1*–*4*). *Clostridioides* (formerly *Clostridium*) species are ubiquitous, gram-positive, spore-forming (most species), and toxin-producing bacteria (*3*). The most well-known toxins, *C. perfringens* α and θ toxins, induce platelet aggregation, diffuse formation of thrombi, cell lysis, and gas gangrene (*5*). Anaerobic bacteria are not only found in the soil or rotting vegetation but also are commensal constituents of the human microbiome, especially in the gastrointestinal tract or genital organs of women (*3*,*6*). Humans are usually infected by direct entry of the bacteria through a wound (*C. tetani*, *C. perfringens*) or by contaminated food (*C. botulae*). However, contamination of a wound by spores is not sufficient to generate the infection because *Clostridioides* spp. need hypoxic and acidic conditions to proliferate. Conditions such as vascular trauma, atherosclerosis, or malignancies may induce tissue hypoxia. Moreover, the liberation of both α and θ toxin, which induce the formation of occlusive thrombi, may increase tissue hypoxia, sometimes leading to gas gangrene formation (*3*,*7*–*9*).

Thus, *Clostridioides* infections are especially known to cause myonecrosis with rapid extension and gas gangrene formation, which, if not treated rapidly, may be fatal. This outcome has largely been described in the context of war wounds, trauma, and surgery (*10*–*12*). Although *C. perfringens* is mostly involved in gas gangrene, other *Clostridioides* subspecies can also be responsible for such infections (*13*).

*Clostridioides* bacteria can also cause primary bacteremia, with or without gas gangrene (*2*,*6*). *Clostridioides* bacteremia are usually fulminant and life-threatening infections. Data focusing on *Clostridioides* bacteremia rely mainly on case reports (*14*–*16*), case series on selected populations (*17*,*18*), or larger epidemiologic series that contain microbiological data but few clinical descriptions (*2*,*19*,*20*). Although *Clostridioides* bacteremia often leads to sudden and massive organ failure requiring transfer to a hospital intensive care unit (ICU), no study has focused on *Clostridioides* bacteremia in the ICU. Therefore, we conducted a multicenter retrospective study of case-patients who were positive for all *Clostridioides* species except *C. difficile* to investigate *Clostridioides* bacteremia in the ICU; we described the clinical spectrum of critically ill patients, ICU admission conditions, microbiological characteristics, and outcomes. We aimed to identify risk factors associated with mortality.

## Methods

### Ethics

This study was approved by an Institutional Review Board (Comité d’Ethique de la Société de Réanimation de Langue Française no. CE-SRLF 18–38) in accordance with the French regulation on noninterventional studies, which waived the need for signed informed consent for patients included in this database. No data allowing identification of the patients included in the study were recorded. The study was conducted in accordance with the Declaration of Helsinki principles.

### Study Population

We retrospectively recorded cases of *Clostridioides* bacteremia in the period July 2003–December 2018 in 15 ICUs in France. Patients were identified by review of ICU medical records and hospital microbiological databases; we selected only cases with >1 positive blood culture for all *Clostridioides* species except *C. difficile*. Blood samples had been collected with specific anaerobic blood culture bottles and incubated in automated systems, in accordance with routine practice (*21*). Anaerobes were identified using the API System (bioMérieux, https://www.biomerieux.com) until 2010; as of 2010, matrix-assisted laser desorption/ionization time-of-flight mass spectrometry methods were used in most of the centers to identify anaerobic bacteria (*3**,**22*). Antimicrobial susceptibility test results of *Clostridioides* species, evaluated by diffusion methods according to guidelines of the Comité de l’Antibiogramme de la Société Française de Microbiologie, were also collected for our study.

We reviewed ICU medical records of selected patients for age, sex, underlying diseases, clinical and biologic symptoms at ICU admission, the need for organ support, antimicrobial drug treatment, and outcome. We recorded Charlson index, simplified acute physiology score (SAPS2), and sequential organ failure assessment (SOFA) scores as previously defined (*23*–*25*). We defined septic shock according to the Sepsis-3 consensus definition (*26*) and hemolysis as low hemoglobin level associated with other hemolysis parameters, such as an increase of lactate dehydrogenase (LDH) or unconjugated bilirubin and reduced haptoglobin levels.

### Statistical Analysis

We described categorical variables as counts and percentages and quantitative variables as median and interquartile range. We estimated mortality rate at 28 days and 90 days after the date of bacteremia, as a binary variable, and examined factors associated with overall survival as a time-to-event endpoint. We defined overall survival as the time between the date of *Clostridioides* bacteremia and the date of death or last follow-up, whichever occurred first. We performed survival analysis using a Cox regression model, estimating hazard ratios (HR) and 95% CIs. We checked the proportional hazards (PH) assumption and the log-linearity assumptions for the models; if the PH assumption was not valid, we used time-dependent coefficient for time-varying effect over time; we used a step function, with time-intervals defined based on the Schoenfeld’s residuals. Factors which were associated to OS with a p<0.1 in univariate analysis were candidates for a multivariate adjusted model. We selected the adjusted model using a backward stepwise procedure, based on the Akaike criterion. All tests were 2-sided; p<0.05 was considered significant. We performed analyses by using the R statistical platform version 3.6.1 (https://www.r-project.org).

## Results

### Clinical and Biologic Manifestations 

**Figure 1:**
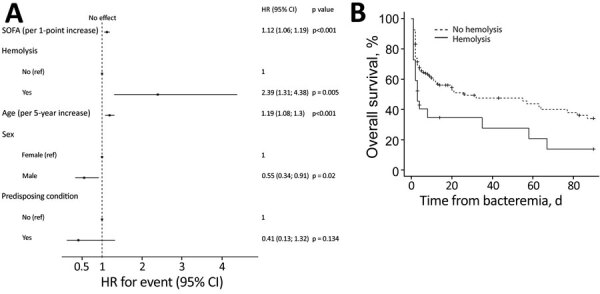
Survival analysis with Cox regression model for patients with *Closteridiodes* bacteremia, France. A) Forest plot of multivariate factors associated with overall survival. Predisposing conditions were trauma or surgery. B) Kaplan-Meier curve of mortality depending on hemolysis. HR, hazard ratio; ref, referent; SOFA, sequential organ failure assessment.

In total, 135 patients with *Clostridioides* bacteremia were identified in 15 ICUs in France during the study period (Table 1); 60% (n = 81) of the patients were men. Median age at diagnosis was 64 years. Most (96%) patients had >1 underlying medical condition; among patients >65 years of age, diabetes mellitus, neoplasms, and chronic obstruction pulmonary disease (COPD) were the most frequent. Thirty-four (26%) patients had an underlying solid tumor from digestive (n = 14, 41%), gynecological (n = 7, 21%), and pancreatic or biliary (n = 4, 12%) origins. Three patients (9%) had urinary tract tumors, 2 (6%) neuroendocrine tumors, 1 (3%) an Ewing tumor, 1 (3%) oral cancer, and 1 (3%) testicular cancer. In all, 94% of tumors were active at the time of the bacteremia. Nineteen (15%) patients had also received diagnoses of hematological malignancies (7 lymphoma, 4 acute lymphoblastic leukemia, 4 myelodysplastic syndrome, 3 acute myeloid leukemia, and 1 myeloproliferative disorder); 3 of those patients had undergone bone marrow transplantation. Thirty-eight patients (28%) had been treated with immunosuppressive agents. In addition, 13 (10%) patients had experienced recent surgery or trauma, and these situations were associated with a better outcome in univariate analysis (Appendix Figure 1). However, this difference was not significant in multivariate analysis (HR 0.41, 95% CI 0.13–1.32; p = 0.13) (Figure 1).

**Table 1:** Global characteristics of patients with *Clotristridium* bacteremia, France*

Characteristic	Baseline population, n = 135	Survived, n -= 65		Died in ICU, n = 70
Age				
Age at diagnosis, median (IQR)	64 (51–75)	62 (50–70)		66 (54–79)
Age at diagnostic >65 y	67 (50)	27 (42)		40 (57)
Sex				
M	81 (60)	43 (66)		38 (54)
F	54 (40)	22 (33)		32 (46)
All underlying conditions	130 (96)	62 (95)		68 (97)
Diabetes mellitus	34 (26)	19 (31)		15 (22)
Neoplasm	34 (26)	17 (27)		17 (25)
Chronic obstructive pulmonary disease	33 (25)	15 (24)		18 (26)
Alcoholism	26 (20)	11 (18)		15 (22)
Heart failure	26 (20)	9 (15)		17 (25)
Hematological malignancy	19 (15)	7 (11)		12 (18)
Liver cirrhosis	13 (10)	4 (6)		9 (13)
Chronic renal failure	10 (8)	8 (13)		2 (3)
Arteriopathy	9 (7)	3 (5)		6 (9)
Autoimmune diseases	7 (5)	4 (6)		3 (4)
HIV	1 (1)	0		1 (1)
Other predisposing conditions				
Surgery or trauma in the previous 15 d	13 (10)	10 (16)		3 (4)
Digestive surgery	10 (77)	7 (70)		3 (100)
Trauma	3 (23)	3 (30)		0
Immunosuppressive agents				
Patients receiving Immunosuppressive agents	38 (28)	18 (28)		20 (29)
Steroids	20 (53)	9 (50)		11 (55)
Chemotherapy	20 (53)	9 (50)		11 (55)
Immunosuppressive drugs	10 (26)	6 (33)		4 (20)

*Data are no. (%) unless otherwise indicated.

*Clostridioides* bacteremia manifested with septic shock at ICU admission in 115 patients (85%), and 26 (19%) patients experienced a cardiac arrest in the ICU (Table 2). Indeed, *Clostridioides* bacteremia causes severe illness, as assessed by high SAPS2 and SOFA scores, high lactate levels, and substantial need for organ supports during an ICU stay. Of note, digestive symptoms were the main symptoms associated with *Clostridioides* bacteremia (62% of patients), whereas myonecrosis represented only 16% of ICU admissions. Acute hemolysis, a distinctive biologic signature of *Clostridioides* bacteremia, was present in 22 (17%) cases (Appendix Table 1). Median hemoglobin level was significantly lower in the hemolysis group (4.9, IQR 3.6–7.0) compared with the rate in patients without hemolysis (10.9, IQR 9.3–12.6; p<0.001). Multiple organ failure, experienced as hepatic cytolysis, acute kidney injury and thrombocytopenia (Table 2), was also common. Of note, aspartate aminotransferase levels were higher than alanine aminotransferase levels, which is commonly found in case of hemolysis. Twenty-seven patients (28%) had <4 × 10^9^ leukocytes/L; 23 (85%) of those had an underlying solid tumor or a hematological malignancy.

**Table 2:** Clinical and biologic characteristics of patients with *Closteridiodes* bacteremia, France*

Characteristic	All patients, n = 135			Survived, n = 65			Died in ICU, n = 70	
	No.	Result		No.	Result		No.	Result
Temperature	75	37 (36–39)		30	38.5 (37.3–39)		45	37 (35.6–38.7)
Clinical manifestations associated with bacteremia at ICU admission								
Septic shock	115	85%		49	75%		66	94%
Digestive symptoms	84	62%		42	65%		42	60%
Acute respiratory failure	41	30%		15	23%		26	37%
Coma	38	28%		6	9%		32	46%
Cardiac arrest	26	19%		2	3%		24	34%
Myonecrosis	21	16%		11	17%		10	14%
Prognostic scores at ICU admission								
Charlson score	135	5 (3–6)		65	5 (2–7)		70	4.5 (3–6)
SAPS2 score	106	63 (44–88)		47	45 (33–57)		59	82 (63–97)
SOFA score	105	10 (7–14)		47	8 (5–10)		58	12 (9–15)
Organ support in ICU								
Vasopressors, n = 132	108	82%		42	66%		66	97%
Mechanical ventilation, n = 133	105	79%		41	63%		64	94%
Renal-replacement therapy, n = 131	44	34%		17	26%		27	41%
Biologic parameters at ICU admission								
Leukocytes, × 10^9^/L	97	9.5 (2.8–17.8)		49	9.5 (6.1–20.2)		48	9.3 (1.6–16.0)
Platelets, × 10^9^/L	93	141 (76–214)		45	143 (73–217)		48	138 (84–206)
Hemoglobin, g/dL	92	10.3 (7.8–12.2)		45	10.2 (8.8–12.2)		47	10.3 (7.0–11.9)
Hemolysis, n = 130	22	17%		6	9%		16	24%
Acute renal failure, n = 107								
KDIGO classification 1	14	13%		10	20%		4	7%
KDIGO classification 2	34	32%		19	38%		15	26%
KDIGO classification 3	59	55%		21	42%		38	67%
Other								
Aspartate aminotransferase, UI/L	80	92 (41–269)		38	71 (41–172)		42	134 (44–346)
Alanine aminotransferase, UI/L	81	54 (25–142)		39	47 (21–125)		42	69 (27–152)
Bilirubin, µmol/L	69	22 (10–45)		34	27 (10.3–52.3)		35	19.5 (10.5–35.7)
Lactatemia, mmol/L	93	5.3 (2.3–8.8)		40	3.2 (1.5–5.2)		53	8 (4.9–12)
pH	93	7.29 (7.13–7.4)		40	7.38 (7.32–7.43)		53	7.18 (7.05–7.31)

*Results are given as percentages or median (interquartile range). ICU, intensive care unit; KDIGO, Kidney Disease: Improving Global Outcomes (https://kdigo.org); SAPS2, simplified acute physiology score (SAPS2); SOFA, sequential organ failure assessment.

### Documentation of Infectious Species

**Figure 2:**
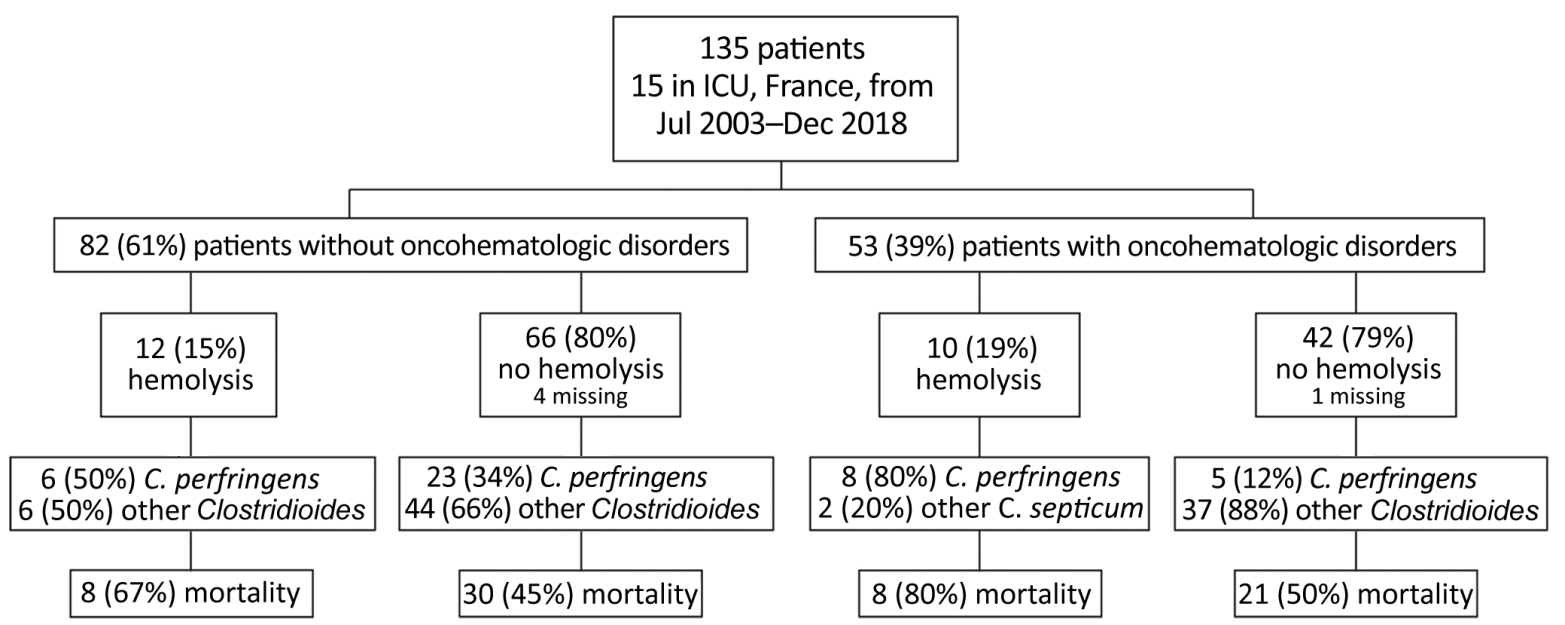
Flowchart of the repartition of *Clostridioides* bacteremia in patients in France according to the presence or absence of hemolysis. Hemolysis was associated with a high mortality rate.

**Figure 3:**
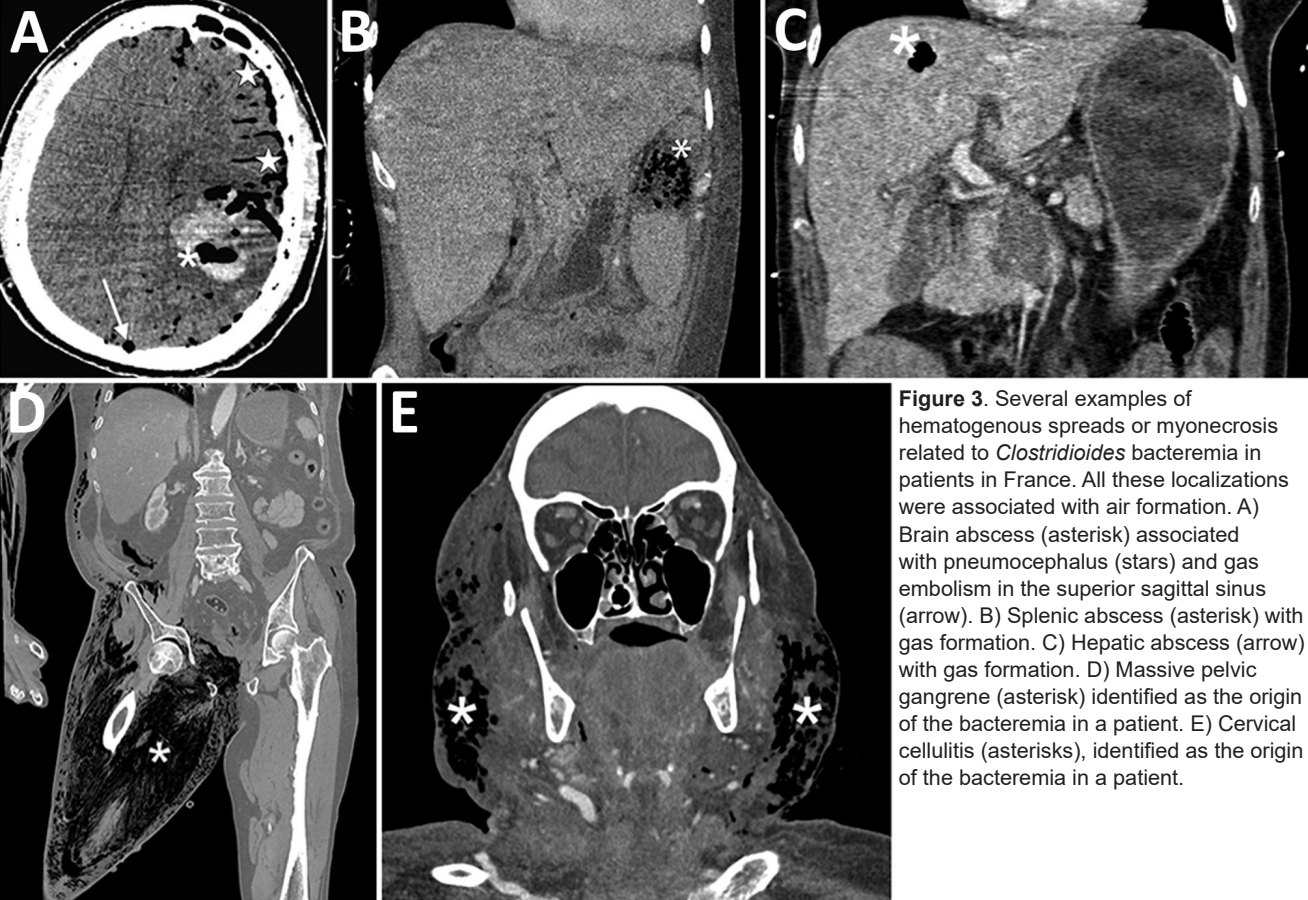
Several examples of hematogenous spreads or myonecrosis related to *Clostridioides* bacteremia in patients in France. All these localizations were associated with air formation. A) Brain abscess associated with pneumocephalus and gas embolism in the superior sagittal sinus. B) Splenic abscess (asterisk) with gas formation. C) Hepatic abscess with gas formation. D) Massive pelvic gangrene identified as the origin of the bacteremia in a patient. E) Cervical cellulitis, identified as the origin of the bacteremia in a patient.

In total, 16 different *Clostridioides* species were identified by blood cultures, including *C. perfringens* in one third of the patients (Table 3; Figure 2). In univariate analysis, documented *C. perfringens* infection was not associated with a worse outcome than other *Clostridioides* species (HR 0.78, CI 95% 0.49–1.23; p = 0.285) (Appendix Figure 2). Blood cultures were mainly performed by peripheral venipuncture (58%), followed by central venous catheter puncture (23%) and arterial catheter puncture (17%). One blood culture revealed *Clostridioides* bacteremia in 87% of cases. Of note, 49 cases of *Clostridioides* bacteremia were polymicrobial bacteremia, yielding the presence of >1 type of bacteria in blood cultures, balanced between gram-negative, gram-positive, and other anaerobic bacteria. Nine patients had both gram-negative and gram-positive bacteria cultures. Hematogenous spread with gas-forming abscess was one particular complication, found in 9 patients and leading to death in 5 in the ICU (Figure 3, panels A–C). As suggested by clinical symptoms, cases of bacteremia were mostly from the gastrointestinal tract (74%), followed by myonecrosis (16%) (Figure 3, panels D–E). In total, 110 (91%) of the patients were treated with antimicrobial drugs in the ICU, and 64 (47%) patients underwent surgery that was mostly gastrointestinal surgery (67% of surgery interventions).

**Table 3:** Characteristics of non–*C. difficile* bacteria in cases of *Clostridioides* bacteremia, France

Characteristic	No. (%) patients					
	All patients, n = 135		Survived, n = 65		Died in ICU, n = 70		
*Clostridium* species							
* Perfringens*	42 (31)		16 (25)		26 (37)		
* Ramosum*	18 (13)		10 (15)		8 (11)		
Any *Clostridioides* sp.	16 (12)		6 (9)		10 (14)		
* Tertium*	14 (10)		9 (14)		5 (7)		
* Clostridiforme*	12 (9)		8 (12)		4 (6)		
* Septicum*	10 (7)		2 (3)		8 (11)		
* Innocuum*	6 (4)		5 (8)		1 (1)		
* Butyricum*	4 (3)		2 (3)		2 (3)		
* Paraputrificum*	3 (2)		2 (3)		1 (1)		
* Baratii*	2 (1)		1 (2)		1 (1)		
* Orbiscindens*	2 (1)		1 (2)		1 (1)		
* Sporogenes*	2 (1)		1 (2)		1 (1)		
* Cadaveris*	1 (1)		0 (0)		1 (1)		
* Novyi*	1 (1)		1 (2)		0 (0)		
* Sordellii*	1 (1)		0 (0)		1 (1)		
* Symbosium*	1 (1)		1 (2)		0 (0)		
No. positive blood cultures for *Clostridioides* spp.							
1	117 (87)		52 (80)		65 (93)		
2	13 (10)		9 (14)		4 (6)		
3	5 (4)		4 (6)		1 (1)		
Other microbes associated with *Clostridioides* bacteremia, n = 49			27		22		
Gram-negative bacteria	33 (67)		20 (74)		13 (59)		
Gram-positive bacteria	24 (49)		12 (44)		12 (55)		
* Candida fungemia*	1 (2)		1 (4)		0 (0)		
Effectiveness of tested antimicrobial drugs against *Clostridioides* species						
Penicillin, n = 84	83 (99)		37 (100)		46 (98)		
Clindamycin, n = 67	46 (69)		22 (67)		24 (71)		
Vancomycin, n = 67	67 (100)		33 (100)		34 (100)		
Metronidazole, n = 84	82 (98)		36 (97)		46 (98)		
Patients receiving drugs	110 (91)		64 (98)		46 (82)		
Beta-lactams	102 (94)		60 (94)		42 (95)		
Amoxicillin/clavulanic acid	9 (9)		6 (10)		3 (7)		
Piperacillin/tazobactam	46 (45)		25 (42)		21 (50)		
Cephalosporins	22 (22)		15 (25)		7 (17)		
Carbapenems	26 (25)		15 (25)		11 (26)		
Aminoglycoside	58 (54)		34 (53)		24 (55)		
Anti–gram positive bacteria	46 (43)		29 (45)		17 (39)		
Metronidazole	39 (36)		26 (41)		13 (30)		
Others	10 (9)		5 (8)		5 (11)		
Missing data	2		0		2		
Origin of bacteremia							
Digestive origin	87 (74)		43 (70)		44 (79)		
Bowel pathology	33 (28)		14 (23)		19 (34)		
Mesenteric ischemia	25 (21)		7 (11)		18 (32)		
Peritonitis	19 (16)		16 (26)		3 (5)		
Pancreatic or biliary origin	10 (9)		6 (10)		4 (7)		
Myonecrosis	19 (16)		11 (18)		8 (14)		
Abscess	8 (7)		5 (8)		3 (5)		
Pneumonia	3 (3)		2 (3)		1 (2)		
Missing data	18		4		14		

Most strains of *Clostridioides* were sensitive to β-lactam drugs. *Clostridioides* species were sensitive to clindamycin in 69% of the cases. Two species (*C. tertium* and *C. septicum*) were resistant to metronidazole. We did not find any association between specific antimicrobial agents used to treat *Clostridioides* bacteremia and mortality (HR 1.01, 95% CI 0.57–1.77; p = 0.977) (Appendix Table 2).

### Outcomes and Mortality Risk Factors

Although *Clostridioides* spp. were for the most part efficiently treated by common antimicrobial drugs, *Clostridioides* bacteremia remained very aggressive and life-threatening; the overall mortality rate at 6 months was 71%. Of 135 patients, 84 died; 70 (52%) of all patients died in the ICU. The 28-day mortality rate was 55% (95% CI 45%–64%), and the 90-day mortality rate was 71% (95% CI 60%–79%). The rapid need for hospitalization after the occurrence of the first symptoms (median days 0, IQR 0–1) highlighted the aggressiveness of *Clostridioides* bacteremia; direct ICU transfer was necessary in most cases (median time between hospitalization and ICU transfer 0 days, IQR 0–2). Median length of stay in ICU was 2 days for patients who did not survive (IQR 1.25–5.75) and 11 days for survivors (IQR 5–23).

In multivariate analysis for overall survival, factors associated with increased risk for death were increasing age (in 5-year increments) (HR 1.19, 95% CI 1.08–1.31; p<0.001), increasing SOFA score (per point) (HR 1.12, 95% CI 1.06–1.19; p<0.001), and presence of hemolysis (HR 2.39, 95% CI 1.31–4.38; p = 0.005). On the other hand, male sex was associated with a reduced risk for death (HR 0.56, 95% CI 0.34–0.91; p = 0.02) (Figure 2).

## Discussion

In our study, we found that *Clostridioides* bacteremia is an aggressive and rapidly life-threatening infection, occurring mainly in patients with underlying conditions. Septic shock with digestive symptoms is the usual manifestation. Despite rapid transfer to the ICU, large use of organ support, and active antimicrobial treatment, *Clostridioides* bacteremia remains highly lethal; 52% of ICU patients died. Massive intravascular hemolysis, associated with fatal complications, should alert clinicians to the possibility of sepsis.

Data on *Clostridioides* bacteremia consist mainly of case reports (*14*–*16*,*27*) or case series that include a small number of patients (*17*,*28*–*30*). Larger publications focusing on anaerobic bacteremia do not provide details on patients’ characteristics and outcomes (*2*,*19*). Furthermore, we could find no previous publications on *Clostridioides* bacteremia in ICU patients, even though anaerobic bacteremia is frequent in this population (*2*,*31*). Our study provides a thorough description of the clinical and biologic characteristics as well as the outcomes of this serious condition.

Our results are consistent with earlier reports; most *Clostridioides* bacteremia cases occur in patients >65 years of age, and prevalence is higher in men (*17*,*29*,*31*). Diseases such as diabetes, chronic kidney disease, heart failure, and COPD, which maintain a baseline degree of organ ischemia and cause chronic organ failure, can lead to *Clostridioides* proliferation and consequently to bacteremia (*1*,*19*,*29*,*31*). Cancer patients or patients with hematological malignancies are also at high risk (*18*,*32*). Chemotherapy-induced cytopenia may result in neutropenic enterocolitis (*33*); this impairment of the natural digestive barrier favors the development of *Clostridioides* bacteremia. Digestive symptoms that have been described as hallmarks of this condition (*1*,*17*,*29*) were frequently associated with *Clostridioides* bacteremia in the ICU. Of note, although *Clostridioides* bacteremia is mostly from digestive origins, myonecrosis was identified as the origin of the bacteremia in 16% of the cases in our study, which is consistent with previous reports (*17*,*18*,*29*,*31*).

Among *Clostridioides* species, *C. perfringens* was more often identified as the source of bacteremia, as previously published (*1*,*19*,*34*). Fifteen other *Clostridioides* species have been identified; distribution is similar to the one described by Leal et al. (*19*). In this study, we chose to exclude *C. difficile* infections because this pathogen is mainly responsible for healthcare-associated digestive infections. *C. difficile* can still present as extradigestive infections; however, few cases of bacteremia have been reported (*35*,*36*).

Data on incidence of anaerobic and *Clostridioides* bacteremia are conflicting. Some authors report an increasing incidence of anaerobic bacteremia since the 1990s, whereas other report decreasing trends (*2*,*6*,*20*,*37*). The incidence of anaerobic bacteremia depend on patients’ age and underlying conditions (especially cancer or cardiovascular illness), and antibiotic selection pressure driven by antimicrobial drug use and environmental conditions. In addition, as suggested by Morris et al. (*38*), blood cultures for anaerobic bacteria may be influenced by patients’ background and clinical symptoms. Indeed, in a recent study evaluating anaerobic bacteremia, 39.7% of the positive blood cultures were considered to be bloodstream infections; the remaining 60.3% were attributed to contaminants (*39*). The evolution of microbiologic techniques, including growing use of automated techniques and matrix-assisted laser desorption/ionization time-of-flight mass spectrometry, might have also influenced the increasing isolation of anaerobic bacteremia. Although we cannot rule out that some infections may have been overestimated, the severity of clinical presentations in our cohort suggests that these cases resulted from true bloodstream infections.

Of note, *Clostridioides* bacteremia can present either as a single microbial bacteremia or as a polymicrobial bacteremia (*2*,*28*,*30*,*31*). *Enterobacteriaceae* were the most commonly associated bacteria, followed by *Staphylococcus* species. Cultures for multiple microbes were positive for *Clostridioides* spp. in 18 patients. Comparable results were also found by Fujita et al. (*17*). *Clostridioides* species are largely susceptible to common antimicrobial drugs, except for clindamycin; susceptibility for clindamycin has been reported as reduced by 73%–96% (*1*,*3*,*5*,*19*,*31*). High susceptibility to penicillin should alert clinicians to rapidly initiate treatment in identified cases of *Clostridioides* bacteremia. Because 94% of the patients in our study received adequate antimicrobial drugs within 24 hours of ICU admission, we were not able to find any statistical association between early initiation of drugs and death. However, in a recent study published by Stabler et al. (*34*), adequate empiric antimicrobial therapy was associated with a better outcome. This result was also highlighted previously by Zahar et al. (*18*).

Mortality rates for *Clostridioides* bacteremia reported in the literature were 15%–48% (*17*–*19*,*28*–*31*), which is lower than the mortality rates reported in our study. However, we focused on critically ill patients. Indeed, Yang (*17*) and Fujita (*31*) revealed that patients who developed shock and required transfer to the ICU had worse outcomes than other patients. In those studies, shock was statistically associated with increased deaths. The prognosis for *Clostridioides* bacteremia patients is also related to underlying conditions that predispose to *Clostridioides* bacteremia and possibilities of therapeutic interventions in addition to prompt and appropriate antimicrobial drugs. As demonstrated by Rechner et al. (*1*), patients who required medical intervention to treat *Clostridioides* bacteremia had lower survival rates than patients who were managed by surgery. Conversely, the presence of massive intravascular hemolysis is a marker of extreme severity, despite appropriate management (*16*,*40*). Hemolysis is induced by *Clostridioides* toxin A (*29*), which hydrolyzes phospholipids in erythrocyte membranes, causing spherocytosis and subsequent intravascular hemolysis. Present in 17% of patients in our cohort, hemolysis is associated with a dramatic increase in mortality rate and remains a strong prognostic factor identified in our study. Finally, *Clostridioides* bacteremia in the ICU is associated with a higher mortality rate than that for classic septic shock in the ICU (*41*,*42*), which makes *Clostridioides* bacteremia a particularly difficult infection to deal with in the ICU.

The first limitation of our study is its retrospective nature and the inherently associated bias, such as missing data and unidentified confounding factors that may have been overlooked in the data collection. However, because of the rarity of *Clostridioides* bacteremia, prospective studies would hardly be feasible. Second, there are no standardized ICU admission policies for these patients, and patient recruitment patterns may have influenced the findings. Given the rapidity of the onset of symptoms and the severity of illness, rapid ICU management was the rule in the participating centers. However, we could not exclude that some patients, because of their advanced age or underlying conditions, were considered too sick for admission to the ICU and may have been denied intensive care.

In conclusion, *Clostridioides* bacteremia is an aggressive infection that often leads to failure of multiple organs, requiring prompt intensive care management. Particular attention should be paid to patients who have underlying conditions and are experiencing hemolysis. Early administration of antimicrobial agents active against *Clostridioides* bacteremia is essential, considering that most *Clostridioides* species are sensitive to β-lactams drugs. Even with prompt and appropriate management, however, *Clostridioides* bacteremia is associated with a high mortality rate in the ICU.

Appendix Additional information about non–*C. difficile Clostridioides* bacteremia in intensive-care patients in France

## References

[1] Rechner PM, Agger WA, Mruz K, Cogbill TH. Clinical features of clostridial bacteremia: a review from a rural area. Clin Infect Dis. 2001;33:349–53. 10.1086/321883 11438901

[2] Gajdács M, Ábrók M, Lázár A, Terhes G, Urbán E. Anaerobic blood culture positivity at a University Hospital in Hungary: A 5-year comparative retrospective study. Anaerobe. 2020;63:102200. 10.1016/j.anaerobe.2020.102200 32247001

[3] Gajdács M, Spengler G, Urbán E. Identification and antimicrobial susceptibility testing of anaerobic bacteria: Rubik’s cube of clinical microbiology? Antibiotics (Basel). 2017;6:25. 10.3390/antibiotics6040025 29112122 PMC5745468

[4] Blairon L, De Gheldre Y, Delaere B, Sonet A, Bosly A, Glupczynski Y. A 62-month retrospective epidemiological survey of anaerobic bacteraemia in a university hospital. Clin Microbiol Infect. 2006;12:527–32. 10.1111/j.1469-0691.2006.01407.x 16700700

[5] Stevens DL, Aldape MJ, Bryant AE. Life-threatening clostridial infections. Anaerobe. 2012;18:254–9. 10.1016/j.anaerobe.2011.11.001 22120198

[6] Gajdács M, Urbán E. Relevance of anaerobic bacteremia in adult patients: A never-ending story? Eur J Microbiol Immunol (Bp). 2020;10:64–75. 10.1556/1886.2020.00009 32590337 PMC7391379

[7] Bryant AE, Chen RY, Nagata Y, Wang Y, Lee CH, Finegold S, et al. Clostridial gas gangrene. I. Cellular and molecular mechanisms of microvascular dysfunction induced by exotoxins of *Clostridium perfringens.* J Infect Dis. 2000;182:799–807. 10.1086/315756 10950774

[8] Bryant AE, Chen RY, Nagata Y, Wang Y, Lee CH, Finegold S, et al. Clostridial gas gangrene. II. Phospholipase C-induced activation of platelet gpIIbIIIa mediates vascular occlusion and myonecrosis in *Clostridium perfringens* gas gangrene. J Infect Dis. 2000;182:808–15. 10.1086/315757 10950775

[9] Ohyama K, Fujimoto M, Nakagomi Y, Ohta M, Yamori T, Kato K. Effect of cyproterone acetate on active and inactive renin secretion in patients with precocious puberty and genetic short stature. Horm Res. 1991;36:216–9. 10.1159/000182166 1840421

[10] Takazawa T, Ohta J, Horiuchi T, Hinohara H, Kunimoto F, Saito S. A case of acute onset postoperative gas gangrene caused by *Clostridium perfringens.* BMC Res Notes. 2016;9:385. 10.1186/s13104-016-2194-0 27488346 PMC4973043

[11] North JP. Clostridial wound infections and gas gangrene; arterial damage as a modifying factor. Surgery. 1947;21:364–72. 20290621

[12] MacLennan JD. The histotoxic clostridial infections of man. Bacteriol Rev. 1962;26:177–276. 10.1128/BR.26.2_Pt_1-2.177-274.1962 14468017 PMC441149

[13] Srivastava I, Aldape MJ, Bryant AE, Stevens DL. Spontaneous *C. septicum* gas gangrene: A literature review. Anaerobe. 2017;48:165–71. 10.1016/j.anaerobe.2017.07.008 28780428

[14] Shen A, Ologun GO, Behm R. Fulminant hepatic failure and fatal cerebral edema following *Clostridium perfringens* bacteremia: case report and review of literature. Cureus. 2017;9:e1714. 10.7759/cureus.1714 29188158 PMC5703590

[15] Wazir M, Jain AG, Nadeem M, Ur Rahman A, Everett G. *Clostridium tertium* bacteremia in a non-neutropenic patient with liver cirrhosis. Cureus. 2019;11:e4432. 10.7759/cureus.4432 31245219 PMC6559675

[16] Simon TG, Bradley J, Jones A, Carino G. Massive intravascular hemolysis from *Clostridium perfringens* septicemia: a review. J Intensive Care Med. 2014;29:327–33. 10.1177/0885066613498043 24019300

[17] Fujita H, Nishimura S, Kurosawa S, Akiya I, Nakamura-Uchiyama F, Ohnishi K. Clinical and epidemiological features of *Clostridium perfringens* bacteremia: a review of 18 cases over 8 year-period in a tertiary care center in metropolitan Tokyo area in Japan. Intern Med. 2010;49:2433–7. 10.2169/internalmedicine.49.4041 21088344

[18] Zahar JR, Farhat H, Chachaty E, Meshaka P, Antoun S, Nitenberg G. Incidence and clinical significance of anaerobic bacteraemia in cancer patients: a 6-year retrospective study. Clin Microbiol Infect. 2005;11:724–9. 10.1111/j.1469-0691.2005.01214.x 16104987

[19] Leal J, Gregson DB, Ross T, Church DL, Laupland KB. Epidemiology of *Clostridium* species bacteremia in Calgary, Canada, 2000-2006. J Infect. 2008;57:198–203. 10.1016/j.jinf.2008.06.018 18672296

[20] De Keukeleire S, Wybo I, Naessens A, Echahidi F, Van der Beken M, Vandoorslaer K, et al. Anaerobic bacteraemia: a 10-year retrospective epidemiological survey. Anaerobe. 2016;39:54–9. 10.1016/j.anaerobe.2016.02.009 26923749

[21] Kirn TJ, Weinstein MP. Update on blood cultures: how to obtain, process, report, and interpret. Clin Microbiol Infect. 2013;19:513–20. 10.1111/1469-0691.12180 23490046

[22] Lévesque S, Dufresne PJ, Soualhine H, Domingo MC, Bekal S, Lefebvre B, et al. A side by side comparison of Bruker Biotyper and VITEK MS: utility of MALDI-TOF MS technology for microorganism identification in a public health reference laboratory. PLoS One. 2015;10:e0144878. 10.1371/journal.pone.0144878 26658918 PMC4689555

[23] Moreno R, Vincent JL, Matos R, Mendonça A, Cantraine F, Thijs L, et al. Working Group on Sepsis-related Problems of the ESICM. The use of maximum SOFA score to quantify organ dysfunction/failure in intensive care. Results of a prospective, multicentre study. Intensive Care Med. 1999;25:686–96. 10.1007/s001340050931 10470572

[24] Charlson ME, Pompei P, Ales KL, MacKenzie CR. A new method of classifying prognostic comorbidity in longitudinal studies: development and validation. J Chronic Dis. 1987;40:373–83. 10.1016/0021-9681(87)90171-8 3558716

[25] Le Gall JR, Lemeshow S, Saulnier F. A new Simplified Acute Physiology Score (SAPS II) based on a European/North American multicenter study. JAMA. 1993;270:2957–63. 10.1001/jama.1993.03510240069035 8254858

[26] Singer M, Deutschman CS, Seymour CW, Shankar-Hari M, Annane D, Bauer M, et al. The third international consensus definitions for sepsis and septic shock (Sepsis-3). JAMA. 2016;315:801–10. 10.1001/jama.2016.0287 26903338 PMC4968574

[27] Fukui M, Iwai S, Sakamoto R, Takahashi H, Hayashi T, Kenzaka T. *Clostridium paraputrificum* bacteremia in an older patient with no predisposing medical condition. Intern Med. 2017;56:3395–7. 10.2169/internalmedicine.8164-16 28943535 PMC5790735

[28] Lopez-Fabal MF, Sanz N, Ruiz-Bastian M, Barros C, Gomez-Garces JL. *Clostridium perfringens* bacteremia, an analysis of 28 cases over 10 years in a university hospital of Madrid [in Spanish]. Enferm Infecc Microbiol Clin. 2018;36:225–8. 10.1016/j.eimc.2017.02.002 28372873

[29] Shindo Y, Dobashi Y, Sakai T, Monma C, Miyatani H, Yoshida Y. Epidemiological and pathobiological profiles of *Clostridium perfringens* infections: review of consecutive series of 33 cases over a 13-year period. Int J Clin Exp Pathol. 2015;8:569–77. 25755747 PMC4348875

[30] Shah M, Bishburg E, Baran DA, Chan T. Epidemiology and outcomes of clostridial bacteremia at a tertiary-care institution. ScientificWorldJournal. 2009;9:144–8. 10.1100/tsw.2009.21 19252754 PMC5823103

[31] Yang CC, Hsu PC, Chang HJ, Cheng CW, Lee MH. Clinical significance and outcomes of *Clostridium perfringens* bacteremia—a 10-year experience at a tertiary care hospital. Int J Infect Dis. 2013;17:e955–60. 10.1016/j.ijid.2013.03.001 23578849

[32] Lark RL, McNeil SA, VanderHyde K, Noorani Z, Uberti J, Chenoweth C. Risk factors for anaerobic bloodstream infections in bone marrow transplant recipients. Clin Infect Dis. 2001;33:338–43. 10.1086/322595 11438899

[33] Duceau B, Picard M, Pirracchio R, Wanquet A, Pène F, Merceron S, et al. Neutropenic enterocolitis in critically ill patients: spectrum of the disease and risk of invasive fungal disease. Crit Care Med. 2019;47:668–76. 10.1097/CCM.0000000000003687 30741755

[34] Stabler S, Titécat M, Duployez C, Wallet F, Loïez C, Bortolotti P, et al. Clinical relevance of *Clostridium* bacteremia: An 8-year retrospective study. Anaerobe. 2020;63:102202. 10.1016/j.anaerobe.2020.102202 32247000

[35] Libby DB, Bearman G. Bacteremia due to *Clostridium difficile*—review of the literature. Int J Infect Dis. 2009;13:e305–9. 10.1016/j.ijid.2009.01.014 19398213

[36] Urbán E, Terhes G, Gajdács M. Extraintestinal *Clostridioides difficile* infections: epidemiology in a university hospital in Hungary and review of the literature. Antibiotics (Basel). 2020;9:16. 10.3390/antibiotics9010016 31906470 PMC7167916

[37] Lassmann B, Gustafson DR, Wood CM, Rosenblatt JE. Reemergence of anaerobic bacteremia. Clin Infect Dis. 2007;44:895–900. 10.1086/512197 17342637

[38] Morris AJ, Wilson ML, Mirrett S, Reller LB. Rationale for selective use of anaerobic blood cultures. J Clin Microbiol. 1993;31:2110–3. 10.1128/JCM.31.8.2110-2113.1993 8370738 PMC265706

[39] Lafaurie M, d’Anglejan E, Donay JL, Glotz D, Sarfati E, Mimoun M, et al. Utility of anaerobic bottles for the diagnosis of bloodstream infections. BMC Infect Dis. 2020;20:142. 10.1186/s12879-020-4854-x 32059701 PMC7023744

[40] Dutton D, Gavrilova N. Massive intravascular hemolysis with organ failure due to *Clostridium perfringens*: evidence of intracytoplasmic *C. perfringens.* Blood. 2013;122:310. 10.1182/blood-2013-01-472407 24032126

[41] Angus DC, Linde-Zwirble WT, Lidicker J, Clermont G, Carcillo J, Pinsky MR. Epidemiology of severe sepsis in the United States: analysis of incidence, outcome, and associated costs of care. Crit Care Med. 2001;29:1303–10. 10.1097/00003246-200107000-00002 11445675

[42] Shankar-Hari M, Phillips GS, Levy ML, Seymour CW, Liu VX, Deutschman CS, et al.; Sepsis Definitions Task Force. Developing a new definition and assessing new clinical criteria for septic shock. JAMA. 2016;315:775–87. 10.1001/jama.2016.0289 26903336 PMC4910392

