# Supplementary material for: Non–C. difficile Clostridioides Bacteremia in Intensive Care Patients, France
Source: Emerg Infect Dis. 2021 Jul;27(7):1840–9. doi: 10.3201/eid2707.203471 (PMC8237868; doi:10.3201/eid2707.203471)
Supplement: Appendix — Additional information about non–C. difficile Clostridioides bacteremia in intensive-care patients in France [file 20-3471-Techapp-s1.pdf]

Article DOI: https://doi.org/10.3201/eid2707.203471

Non–*C. difficile Clostridioides* Bacteremia in Intensive Care Patients, France

Appendix

Appendix Table 1. Biologic parameters of 22 intensive-care patients with bacteremia experiencing hemolysis*

Patient no.	Hemolysis	Hemoglobin, g/dL	LDH, UI/L	ASAT, UI/L	ALAT, UI/L	Bilirubin, µmol/L	*Clostridioides* species
1	Yes	13.1	1,834	46	19	35.1	*C. perfringens*
4	Yes	5.3	HI	HI	HI	HI	*C. perfringens*
5	Yes	1.7	HI	HI	HI	HI	*C. perfringens*
6	Yes	1.2	HI	HI	HI	14	*C. perfringens*
13	Yes	2.8	510	–	–	63	*C. perfringens*
16	Yes	3.4	2,905	2,634	1,608	12.2	*C. septicum*
17	Yes	HI	HI	HI	HI	HI	*C. perfringens*
26	Yes	3.3	5,033	402	32	112	*C. perfringens*
27	Yes	7.1	–	137	26	9	*C. septicum*
28	Yes	3.8	–	38	47	8.6	*C. orbiscindens*
31	Yes	8.2	469	132	93	7.6	*C. perfringens*
32	Yes	4.0	446	46	16	14	*C. novyi*
34	Yes	8.5	3,788	878	1,094	37.4	*C. septicum*
35	Yes	HI	HI	HI	HI	HI	*C. perfringens*
53	Yes	8.8	4,501	1,644	588	10	*C. innocuum*
60	Yes	3.8	630	58	17	53	*C. perfringens*
74	Yes	4.9	3,830	50	30	72	*C. perfringens*
84	Yes	HI	HI	HI	HI	HI	*C. ramosum*
94	Yes	5.9	–	158	46	–	*C. perfringens*
96	Yes	6.9	–	1,696	476	11	*C. baratii*
115	Yes	6.0	–	–	–	25	*C. perfringens*
132	Yes	4.6	–	–	–	–	*C. perfringens*

*Biologic parameters were not measurable for 3 patients due to hemolysis interferences. ALAT, alanine aminotransferase; ASAT, aspartate aminotransferase; HI, hemolysis interference; LDH, lactate dehydrogenase.

Appendix Table 2. Univariate analysis by antimicrobial agents of patients who died in study of non-*C. difficile Clostridiodies* bacteremia, France*

Category	HR (95% CI)	p value
Patients receiving antimicrobial drugs	1.01 (0.57–1.77)	0.977
Antimicrobial drugs		
Beta-lactams	0.88 (0.32–2.43)	0.799
Aminoglycoside	1.24 (0.73–2.08)	0.427
Anti–gram-positive bacteria	0.79 (0.47–1.34)	0.379
Metronidazole	0.81 (0.47–1.41)	0.461
Others	1.31 (0.56–3.06)	0.537

*HR, hazard ratio.

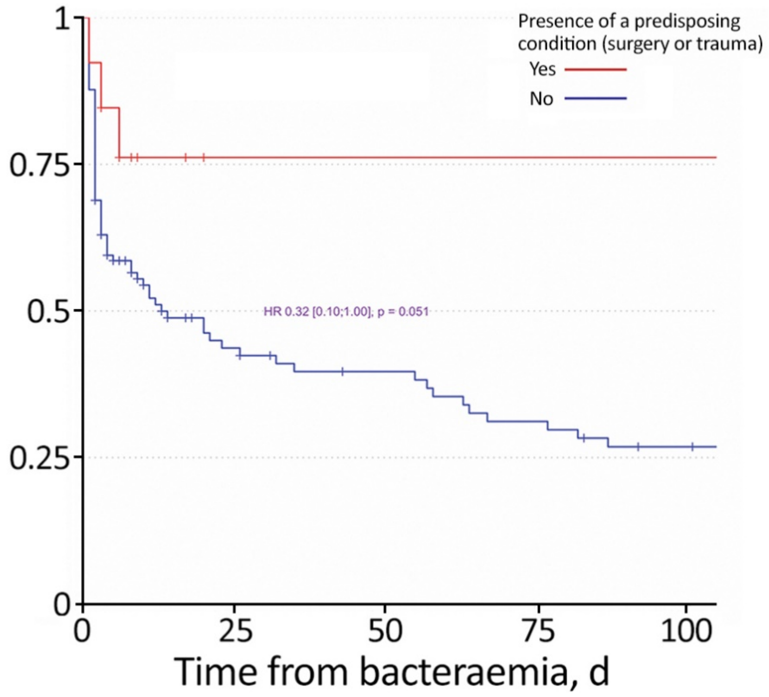

Appendix Figure 1. Kaplan-Meier curve depending on the presence of predisposing conditions. Patients with a recent history of surgery or trauma had a better, but not significant, prognostic compared to other patients. HR, hazard ratio.

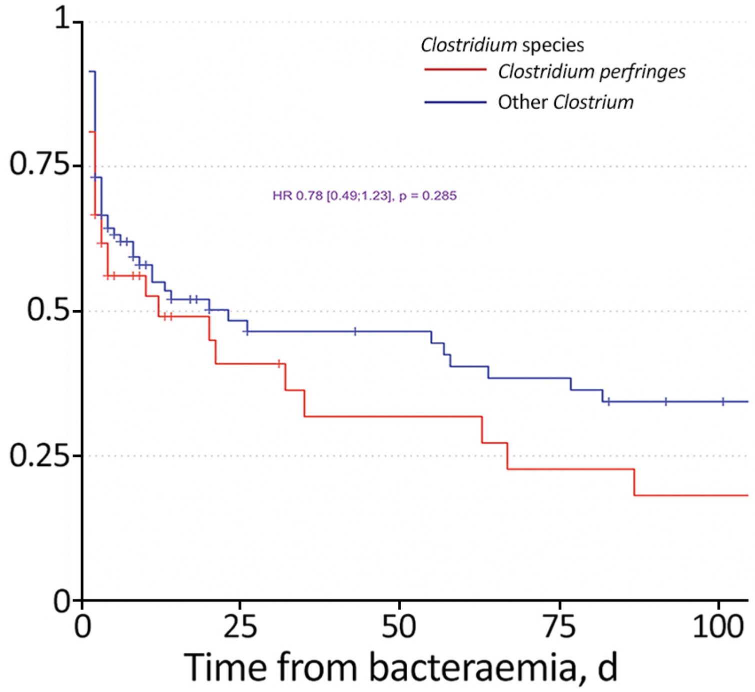

Appendix Figure 2. Kaplan-Meier curve depending on *Clostridioides* species.
